# Cerebral Venous Thrombosis following Sacituzumab Govitecan in Metastatic Breast Cancer: A Case Report

**DOI:** 10.1159/cro/abzag033

**Published:** 2026-08-25

**Authors:** Ahmed M. Abdalhadi, Fadel Alqatati, Asiya Thakur, Ali Ali Alsuyihili, Muhammed Hajmusa, Mohammad Elhissi, Nabil E. Omar

**Affiliations:** a Department of Medical Oncology, National Center for Cancer Care and Research, Hamad Medical Corporation, Doha, Qatar; b Department of Internal Medicine and Medical Education, Hamad Medical Corporation, Doha, Qatar; c Department of Radiology, Hamad General Hospital, Hamad Medical Corporation, Doha, Qatar; d Department of Pharmacy, National Center for Cancer Care and Research, Hamad Medical Corporation, Doha, Qatar; e Health Sciences Program, Clinical and Population Health Research, College of Pharmacy, Qatar University, Doha, Qatar

**Keywords:** Cerebral venous thrombosis, Metastatic breast cancer, Sacituzumab govitecan, Antibody-drug conjugate, Case report

## Abstract

**Introduction:**

Cerebral venous thrombosis (CVT) is an uncommon but potentially serious cerebrovascular disorder that may occur in the setting of malignancy and other prothrombotic states. Prompt diagnosis with venous neuroimaging and early anticoagulation are central to management.

**Case Presentation:**

We report the case of a 33-year-old woman with metastatic hormone receptor-positive/HER2-negative breast cancer who developed intermittent right-sided tremor-like episodes shortly after her third cycle of sacituzumab govitecan. Magnetic resonance imaging demonstrated thrombosis involving the superior sagittal, right transverse, and right sigmoid sinuses, with extension into the visualized right internal jugular vein, and showed no intracranial metastases. She was treated initially with therapeutic enoxaparin, followed by apixaban, with early radiologic recanalization on follow-up MRV. Sacituzumab govitecan was continued after multidisciplinary discussion because a partial response of metastatic disease had been observed.

**Conclusion:**

This case highlights the need to consider CVT in patients with cancer who present with acute neurologic symptoms during systemic therapy and highlights diagnostic and therapeutic challenges in this context. We found no clearly indexed prior published case of CVT temporally associated with sacituzumab govitecan.

## Plain Language Summary

Cerebral venous thrombosis (CVT) is a rare but serious type of blood clot in the veins that drain blood from the brain. It can be present with symptoms such as headache, seizures, or abnormal body movements and requires urgent diagnosis and treatment. We describe a 33-year-old woman with metastatic breast cancer who developed intermittent involuntary movements on the right side of her body shortly after her third cycle of sacituzumab govitecan. Brain imaging confirmed extensive CVT, while showing no evidence of brain metastases. She was treated promptly with anticoagulation, first with enoxaparin and then with apixaban, and follow-up imaging showed early recanalization. Because her metastatic disease had shown a partial response, sacituzumab govitecan was continued after multidisciplinary review. This case is clinically important because it shows that new neurologic symptoms during cancer treatment should not automatically be attributed to brain metastases, and that CVT must remain an important differential diagnosis. It also adds to the limited literature on unusual thrombotic events occurring during antibody-drug conjugate therapy, although a definite causal relationship with sacituzumab govitecan cannot be confirmed because advanced cancer itself is a strong prothrombotic condition.

## Introduction

Cerebral venous thrombosis (CVT) is an uncommon but important cause of stroke, accounting for approximately 0.5%–3% of all strokes [1]. Its clinical presentation is heterogeneous and may include headache, seizures, focal neurologic deficits, or altered mental status [1, 2]. Diagnosis relies on venous neuroimaging, most commonly magnetic resonance imaging with venography (MRI/MRV) or computed tomography with venography [1, 2]. Guidelines from the American Heart Association/American Stroke Association (AHA/ASA) and the European Stroke Organization (ESO) endorse initial parenteral anticoagulation for acute CVT management, followed by oral anticoagulation for secondary prevention [1, 2]. Emerging evidence supports the use of direct oral anticoagulants (DOACs) in CVT, informed by randomized trials and observational cohorts, albeit with persistent knowledge gaps in subpopulations such as those with malignancy [3, 4].

Cancer is a well-recognized prothrombotic state and is associated with an increased risk of CVT [5]. Potential mechanisms include tumor-associated hypercoagulability, direct venous sinus invasion or compression, and treatment-related factors [6, 7].

Sacituzumab govitecan, an antibody-drug conjugate targeting Trop-2, has demonstrated efficacy in phase 3 trials for metastatic triple-negative breast cancer (ASCENT) and hormone receptor-positive/HER2-negative metastatic breast cancer (TROPiCS-02), improving progression-free survival (PFS) and overall survival (OS) relative to standard chemotherapy [8–10]. Thrombotic complications are recognized in oncologic and therapeutic contexts, yet uncommon sites such as CVT pose unique diagnostic and management dilemmas. This report delineates a case of extensive CVT temporally linked to sacituzumab govitecan in a young woman with metastatic breast cancer, adhering to the CARE guidelines for case reporting [11].

## Case Presentation

A 33-year-old woman with a background of stage IV metastatic breast cancer presented to our hospital with a 1-day history of right-sided involuntary movements. She experienced three brief, self-limited episodes of right-sided tremor-like movements involving the arm and leg, each causing a near-fall without loss of awareness. She returned fully to baseline between episodes, which resolved spontaneously within 1 day and did not recur during hospitalization. The semiology was compatible with a possible focal aware motor seizure, although this was not confirmed. EEG was not performed, and no antiseizure therapy was given. She had received her third cycle of sacituzumab govitecan 1 day prior to presentation. Two days before admission, she attended the urgent care department with headache, nausea, and multiple episodes of vomiting. There was no fever or neck stiffness; blood tests were unremarkable for infection, and a viral panel was negative. She improved with analgesia and antiemetics and was discharged. The clinical timeline is summarized in Table 1.

**Table 1.** Clinical timeline

Relative time	Setting	Key events
Day 2	Urgent care	Headache, nausea, multiple vomiting episodes; no fever or neck stiffness; blood tests unremarkable for infection; viral panel negative; improved with analgesia and antiemetics; discharged
Day 1	Chemotherapy	Third cycle of sacituzumab govitecan administered
Day 0	Hospital presentation	Three brief right-sided tremor-like episodes involving the upper and lower extremities, accompanied by near falls without loss of consciousness; complete recovery between episodes; normal vital signs and no focal neurologic deficits
Day 0–1 (inpatient)	Inpatient workup	MRI brain with contrast: extensive cerebral venous sinus thrombosis (Fig. 1a–d); cervical Doppler: patent internal jugular and subclavian veins; echocardiography: no intracardiac thrombus; staging PET-CT: partial morpho-metabolic response with limited progression in selected sites
Day 5	​	MRV confirmed CVT and demonstrated early recanalization of the right transverse sinus (Fig. 1e)
Discharge	Inpatient → outpatient	Enoxaparin for 3 days and then apixaban; sacituzumab govitecan continued after multidisciplinary discussion with primary oncologist

The patient had been diagnosed with bilateral invasive ductal carcinoma of the breast in 2022. Immunohistochemical analysis demonstrated positive expression of estrogen receptors, progesterone receptors, and E-cadherin, with negative HER2 status. She was initially staged as T3N1Mx on the left and T1N0Mx on the right. She received neoadjuvant chemotherapy comprising four cycles of epirubicin and cyclophosphamide followed by 12 weeks of paclitaxel, before undergoing wide local excision with sentinel lymph node biopsy of the right breast and mastectomy with axillary clearance of the left breast. Bilateral radiation therapy was administered, and she was subsequently commenced on goserelin and ribociclib. A follow-up PET scan demonstrated bone and liver metastases. Capecitabine was initiated with an initial response; however, subsequent imaging revealed disease progression with additional metastases to bone, liver, lung, and lymph nodes. Everolimus and exemestane produced a poor response. Sacituzumab govitecan was initiated as a later-line treatment.

On examination, vital signs were within normal limits, and neurological examination revealed no focal deficits. Blood tests showed no leukocytosis or elevation of inflammatory markers; renal function and electrolytes were normal. Targeted thrombophilia testing did not identify an inherited or acquired prothrombotic disorder. Lupus anticoagulant, anticardiolipin antibodies, and anti-β2-glycoprotein I antibodies were negative; protein S and antithrombin activities were normal, and protein C activity was elevated, excluding deficiency. The activated protein C resistance screen was negative by the laboratory’s factor V Leiden threshold (ratio 0.78; positive cutoff ≤0.70). Overall, no definitive thrombophilia was demonstrated.

Urgent contrast-enhanced MRI of the brain demonstrated increased signal on T1- and T2-weighted sequences with post-contrast filling defects in the superior sagittal sinus, right transverse sinus, right sigmoid sinus, and the visualized right internal jugular vein, consistent with cerebral venous sinus thrombosis (Fig. 1a–d). No intracranial metastatic lesions were identified. Patchy marrow signal changes and enhancement of the calvarial bones, clivus, and cervical vertebrae were noted and considered likely to represent metastatic lesions.

**Fig. 1.**
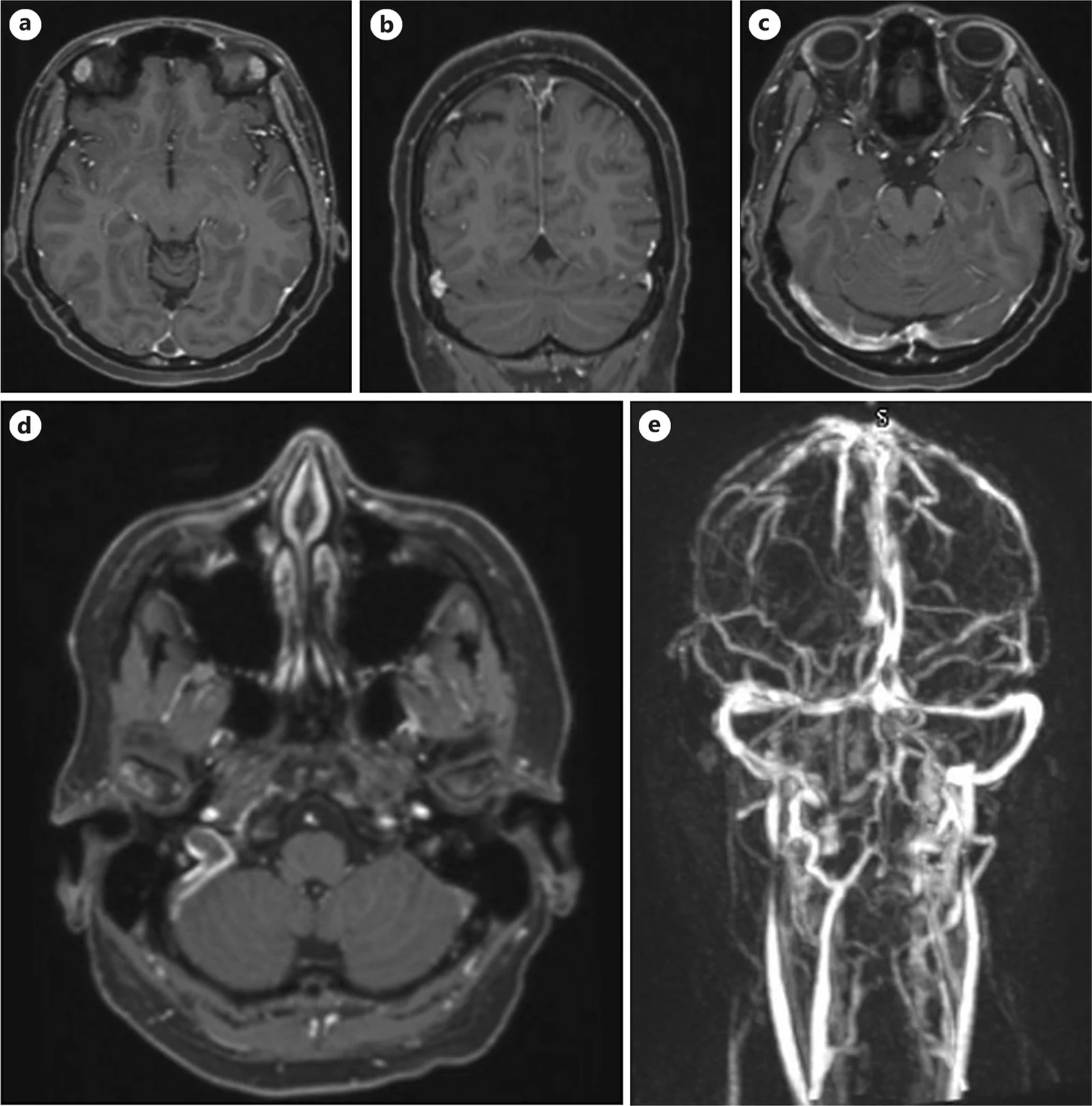
Contrast-enhanced brain MRI and follow-up MRV findings in CVT. **a** Axial T1-weighted post-contrast image demonstrating a filling defect within the superior sagittal sinus, consistent with thrombosis (empty delta sign). **b** Coronal T1-weighted post-contrast image demonstrating a filling defect within the superior sagittal sinus. **c** Axial T1-weighted post-contrast image demonstrating thrombosis of the right sigmoid sinus. **d** Axial T1-weighted post-contrast image demonstrating thrombosis extending into the visualized right internal jugular vein. **e** Brain MRV obtained 5 days after initiation of anticoagulation demonstrating early recanalization of the right transverse sinus thrombosis.

The neurology team advised initiation of therapeutic-dose enoxaparin for 3 days, with subsequent transition to a DOAC. Cervical Doppler ultrasound demonstrated patent internal jugular and subclavian veins. Echocardiography showed normal cardiac function without intracardiac thrombi. A staging PET-CT scan revealed partial morpho-metabolic response in the lung, lymph node, bone, and liver metastases compared with the prior scan, alongside minimal progression in retroperitoneal, para-aortic, and retrocaval lymph nodes and moderate progression of a lesion in the left hepatic lobe. The patient was initially commenced on therapeutic anticoagulation with enoxaparin at a dose of 1 mg/kg administered twice daily, after which she was transitioned to apixaban after 3 days. Magnetic resonance venography of the brain, performed 5 days after the initiation of therapy, demonstrated early recanalization of the right transverse sinus thrombosis (Fig. 1e).

The patient was discharged on therapeutic anticoagulation with apixaban. After multidisciplinary discussion with her primary oncologist, sacituzumab govitecan was continued given the partial metastatic response, with heightened vigilance for thrombotic complications.

After completing seven cycles over approximately 6 months, the disease progressed. She died 1 month later due to respiratory failure secondary to pulmonary metastases. The PFS was 7 months, and the OS was 8 months.

## Discussion

CVT warrants consideration in individuals presenting with new headache, seizures, or focal neurologic deficits, and guidelines advocate prompt venous neuroimaging upon clinical suspicion [1, 2]. Anticoagulation forms the therapeutic cornerstone, commencing with heparin and transitioning to oral agents; duration is guided by provoking factors and recurrence risk [1, 2].

Malignancy is a prominent prothrombotic risk factor. In a case-control study of 594 CVT cases and 6,278 controls, cancer was present in 8.9% of cases and 2.5% of controls and was associated with an adjusted odds ratio of 4.86 (95% confidence interval, 3.46–6.81); the corresponding estimate for solid tumors was 3.07 (95% confidence interval, 2.03–4.65) [5]. That study did not stratify solid tumors by metastatic status and therefore did not provide a metastatic-specific risk estimate. US inpatient data indicate that approximately 6% of primary CVT hospitalizations involve active malignancy, with breast cancer notable among solid tumor diagnoses [12]. Pathogenic mechanisms encompass hypercoagulability, vascular involvement by tumors, and treatment-related contributions [6, 7].

Prior reports have documented CVT in breast cancer across various treatment settings, including palliative regimens where CVT mimicked metastatic disease, and in association with tamoxifen or ribociclib [13–15]. These cases underscore the importance of avoiding presumptive attribution to metastasis without venous imaging evaluation, particularly in the absence of parenchymal lesions [7, 13].

Phase 3 data confirm the superiority of sacituzumab govitecan over standard chemotherapy for both PFS and OS in specified metastatic breast cancer subtypes [8–10]. The USA prescribing information identifies pulmonary embolism as a fatal adverse event in the TROPiCS-02 trial (0.4%) [16]. Although thrombotic events are monitored in treated populations, a causal association between sacituzumab govitecan and CVT cannot be established from the present single case [16]. Two independent FAERS disproportionality analyses evaluated 1,072 cases reported from the second quarter of 2020 through the fourth quarter of 2022 and 2,069 primary-suspect reports from April 2020 through March 2023, respectively [17, 18]. Neither analysis reported CVT among the significant preferred-term disproportionality signals. Thus, there is no established FAERS signal for CVT with sacituzumab govitecan. The present case is therefore hypothesis-generating and adds detailed clinical and radiologic characterization of a potentially very rare event; spontaneous-reporting analyses cannot establish incidence or causality and remain susceptible to underreporting and confounding [17, 18].

In the current case, CVT followed the third sacituzumab govitecan cycle, preceded by a prodrome of headache and vomiting. Nonetheless, the baseline prothrombotic milieu, advanced multi-organ metastases and prior thrombogenic therapies, renders causality indeterminate; cancer-associated thrombosis remains a plausible primary etiology [5–7].

With respect to anticoagulation, guidelines endorse acute heparin followed by oral anticoagulation [1, 2]. The RE-SPECT CVT randomized trial demonstrated the equivalence of dabigatran to warfarin following initial parenteral therapy [3]. Observational data suggest DOAC comparability to warfarin in clinical practice [4]. Apixaban-specific evidence in CVT is limited; a multicenter cohort study reported outcomes similar to vitamin K antagonists but excluded patients with active cancer, restricting its oncologic applicability [19]. The regimen employed, enoxaparin transitioning to apixaban, aligns with evolving DOAC evidence in CVT and necessitates individualized assessment in the setting of malignancy [3, 4].

This case reinforces several clinical principles: intermittent unilateral movements following chemotherapy administration, particularly with a prodromal symptom phase, warrant CVT evaluation alongside seizure and metastasis in patients with advanced cancer [1, 2]. In this patient, three brief unilateral motor episodes with preserved awareness and complete interictal recovery were clinically compatible with possible focal aware motor seizures. However, the events resolved spontaneously within 1 day, no further episodes occurred, and EEG was not performed; therefore, an epileptic mechanism could not be confirmed. No antiseizure therapy was administered. Because the primary diagnosis was established by MRI/MRV, the absence of EEG limits classification of the transient motor manifestation but does not affect the diagnosis of CVT. MRI/MRV enabled diagnosis in the absence of parenchymal metastases, affirming guideline-recommended imaging strategies [1, 2]. Thrombotic events occurring during sacituzumab govitecan therapy merit structured reporting within the framework of ongoing pharmacovigilance [16]. Clinicians should remain alert to CVT when patients with advanced breast cancer receiving sacituzumab govitecan develop new headache or focal neurologic symptoms, while recognizing that the present case does not establish drug causality.

To further evaluate the potential relationship between sacituzumab govitecan and CVT, a structured drug-event causality assessment was performed using the Naranjo Adverse Drug Reaction Probability Scale and the World Health Organization-Uppsala Monitoring Centre (WHO-UMC) system. The Naranjo scale yielded a score of 3, indicating a possible adverse drug reaction. This assessment was based on the temporal association between drug administration and symptom onset, objective radiologic confirmation of CVT, and targeted thrombophilia testing that did not identify a definitive alternative disorder. However, factors such as the lack of drug rechallenge, absence of dose-response data, and the presence of confounding risk factors, including advanced malignancy and prior systemic therapies, limited the strength of attribution [20].

According to the WHO-UMC causality system, the association is best classified as “possible,” defined as an event with a reasonable temporal relationship to drug exposure that could also be explained by underlying disease or other risk factors. In this case, the patient’s metastatic breast cancer and cumulative exposure to potentially thrombogenic oncologic therapies represent significant alternative contributors [21].

Overall, while the temporal proximity suggests a potential association, definitive causality between sacituzumab govitecan and CVT cannot be established. This case underscores the importance of continued pharmacovigilance and systematic reporting of rare thrombotic events associated with antibody-drug conjugates to enhance post-marketing safety data.

## Conclusion

Extensive CVT was diagnosed in a young woman with metastatic breast cancer presenting with transient right-sided movements following sacituzumab govitecan administration. This case highlights the importance of CVT consideration in oncologic neurologic presentations without parenchymal metastases and underscores the need for urgent MRI/MRV [1, 2]. While temporal association exists, causality remains indeterminate in this prothrombotic oncologic context, advocating for enhanced surveillance, structured adverse event reporting, and further case accrual.

## Statement of Ethics

The study protocol was reviewed and approved by the Medical Research Center, Hamad Medical Corporation, Doha, Qatar, Approval No. MRC-04-25-087. Written informed consent was obtained from the patient for publication of this case report and accompanying images. The CARE Checklist for this case report is available as online supplementary material (for all online suppl. material, see https://doi.org/10.1159/cro/abzag033).

## Conflict of Interest Statement

The authors declare that they have no conflicts of interest relevant to this work.

## Funding Sources

The authors received no funding for this work.

## Author Contributions

Ahmed M. Abdalhadi: conceptualization, investigation, data curation, visualization, drafting of the manuscript, and project administration. Fadel Alqatati: data curation, literature review, and critical revision of the manuscript. Asiya Aqeel Thakur: literature review, validation, and manuscript review and editing. Ali Ali Alsuyihili: data collection, validation, and manuscript review and editing. Muhammed Hajmusa: supervision, methodology, critical revision, and manuscript review and editing. Mohammad Elhissi: radiologic interpretation, visualization, validation, and manuscript review and editing. Nabil Omar: pharmacologic review, methodology, validation, and manuscript review and editing. All authors made substantial contributions to the work, approved the final version of the manuscript, and agreed to be accountable for all aspects of the work.

## Data Availability

All data supporting the findings of this case report are contained within the manuscript. No additional datasets were generated or analyzed beyond those reported here. Further inquiries can be directed to the corresponding author.
